# Supplementary material for: Discovery and Characterization of a Chemical Probe Targeting the Zinc-Finger Ubiquitin-Binding Domain of HDAC6
Source: J Med Chem. 2023 Jul 27;66(15):10273–88. doi: 10.1021/acs.jmedchem.3c00314 (PMC10424181; doi:10.1021/acs.jmedchem.3c00314)

SUPPORTING INFORMATION

Discovery and characterization of a chemical probe targeting the zinc-finger ubiquitin-binding domain of HDAC6

Rachel J. Harding[1,2†], Ivan Franzoni[3†§], Mandeep K. Mann[1†], Magdalena M. Szewczyk[1†], Bijan Mirabi[3†], Renato Ferreira de Freitas[1], Dominic D. G. Owens[1], Suzanne Ackloo[1], Alexej Scheremetjew,[3] Kevin A. Juarez-Ornelas[3], Randy Sanichar[3], Rachel J. Baker,[3] Christian Dank[3], Peter J. Brown[1], Dalia Barsyte-Lovejoy[1,2], Vijayaratnam Santhakumar[1], Matthieu Schapira[1,2], Mark Lautens[3], Cheryl H. Arrowsmith*[1,4]*

[1] Structural Genomics Consortium, University of Toronto, Ontario, M5G 1L7, Canada

[2] Department of Pharmacology & Toxicology, University of Toronto, Toronto, Ontario, M5S 1A8, Canada

[3] Davenport Research Laboratories, Department of Chemistry, University of Toronto, Toronto, Ontario, M5S 3H6, Canada

[4] Princess Margaret Cancer Centre and Department of Medical Biophysics, University of Toronto, Toronto, Ontario, M5G 1L7, Canada

[§] Author has updated affiliation: Valence Discovery Inc., 6666 Rue St-Urbain, Suite 200, Montreal, Quebec, H2S 3H1, Canada

[†] These authors contributed equally

* Corresponding authors – Mark Lautens - Mark.Lautens@utoronto.ca, Cheryl H. Arrowsmith - Cheryl.Arrowsmith@uhnresearch.ca

Contents of Supporting Information:

S2 - Table S1. Affinity of HDAC6 proteins for ubiquitin and ISG substrates used in this study
S3 - Table S2. Data collection and refinement statistics for HDAC6 co-crystal structures.
S4 - Figure S1. Omit map (σ2) of compounds from HDAC6-UBD crystal structures determined during structure-based optimization.
S5 - Figure S2. Biophysical characterization of 25 and 32.
S6 - Figure S3. Inhibitory activity of 25.
S7 - Figure S4. Compound 25 does not inhibit HDAC6 catalytic activity in cells.
S8 - Synthesis of the biotinylated compound (33)
S12 - HPLC traces of the compounds 9-32

Table S1. Affinity of HDAC6 proteins for ubiquitin and ISG substrates used in this study

Substrate	HDAC6 ZnF-UBD		HDAC6 ZnF-UBD R1155A Y1184A	Full-Length HDAC6
	FP K_D (μM)[a]	SPR K_D (μM)[b]	SPR K_D (μM)[b]	SPR K_D (μM)[b]
**RLRGG peptide**	NT	11 ± 4.3	NT	40 ± 25
**LRLRGG peptide**	NT	3.8 ± 0.72	NT	10 ± 6.9
**FITC-RLRGG peptide**	0.22 ± 0.013	0.33 ± 0.047	NB	0.23 ± 0.060
**FITC-LRLRGG peptide**	0.039 ± 0.022	0.33 ± 0.15	NB	0.77 ± 0.29
**Ubiquitin**	NT	3.2 ± 0.24	NB	1.0 ± 0.11
**ISG15**	NT	9.3 ± 0.60	NB	11 ± 2.3

[a]K_D determination experiments were performed with N=2 and the values are presented as mean ± SD, reported to 2 significant figures. [b]K_D determination experiments were performed with N=3 and the values are presented as mean ± SD. NT= not tested. NB= no binding.

Table S2. Data collection and refinement statistics for HDAC6 co-crystal structures.

**Compound**<br>**PDB ID**	**9**<br>**8G43**	**15**<br>**8G44**	**25**<br>**8G45**
Space group	$P2_12_12_1$	$P2_12_12_1$	$P2_12_12_1$
a,b,c [Å]	40.87, 44.04, 55.87	40.95, 44.43, 56.13	40.61, 44.91, 55.70
α,β,γ [°]	90.00, 90.00, 90.00	90.00, 90.00, 90.00	90.00, 90.00, 90.00
Resolution limits [Å]	29.98-1.55 (1.58-1.55)	33.08-1.55 (1.58-1.55)	55.70-1.62 (1.65-1.62)
Rmerge	0.042 (0.254)	0.041 (0.216)	0.053 (0.551)
I/sigma	30.7 (7.9)	28.2 (7.9)	14.6 (3.4)
Rpim	0.018 (0.112)	0.025 (0.139)	0.030 (0.317)
CC1/2	1.000 (0.974)	0.999 (0.964)	0.999 (0.923)
Completeness [%]	99.9 (100.0)	97.9 (94.6)	99.7 (99.2)
Multiplicity	6.6 (6.1)	6.7 (6.2)	7.1 (7.5)
No. Reflections used/free	15157/760	14708 (712)	12739/681
Rwork/Rfree	0.161/0.177	0.166/0.198	0.160/0.190
No. Atoms/B-factors [$Å^2$]			
Protein	787/12.4	771/9.7	771/31.1
Ligand	21/14.0	27/8.6	30/34.8
Water	89/22.3	106/19.0	56/39.6
Rmsd bond angle [°]/Rmsd bond length [Å]	1.850/0.028	1.975/0.016	1.689/0.011
Avg B-factors [$Å^2$]	13	11	31
Molprobity Ramachandran favored/outliers [%]	97/2	93/1	95/1

Values for outer shell in brackets. Omit maps for each ligand are shown in **Figure S1**.

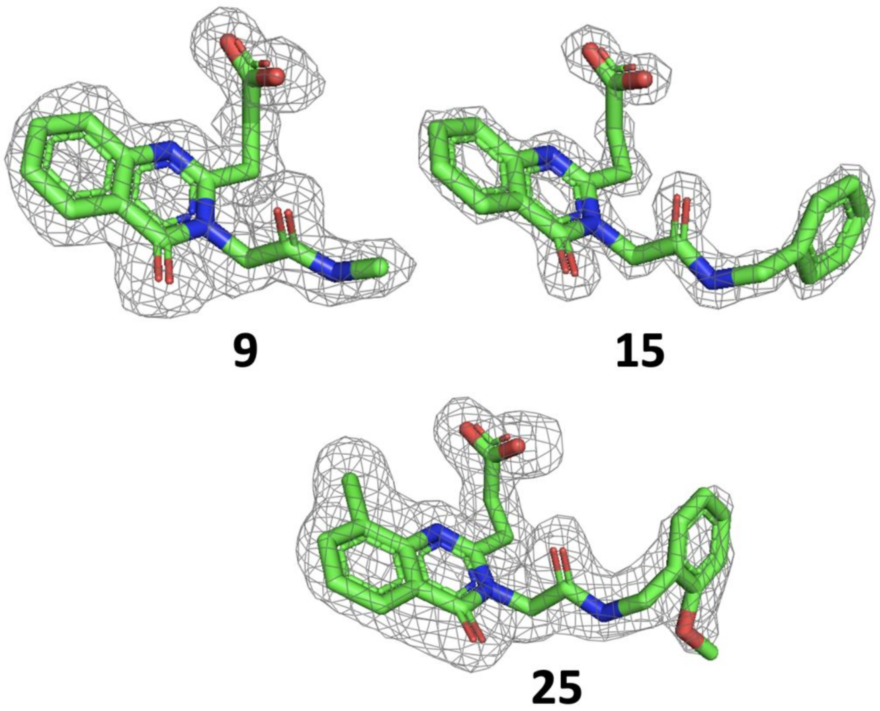

Figure S1. Omit map (σ2) of compounds from HDAC6-UBD crystal structures determined during structure-based optimization.

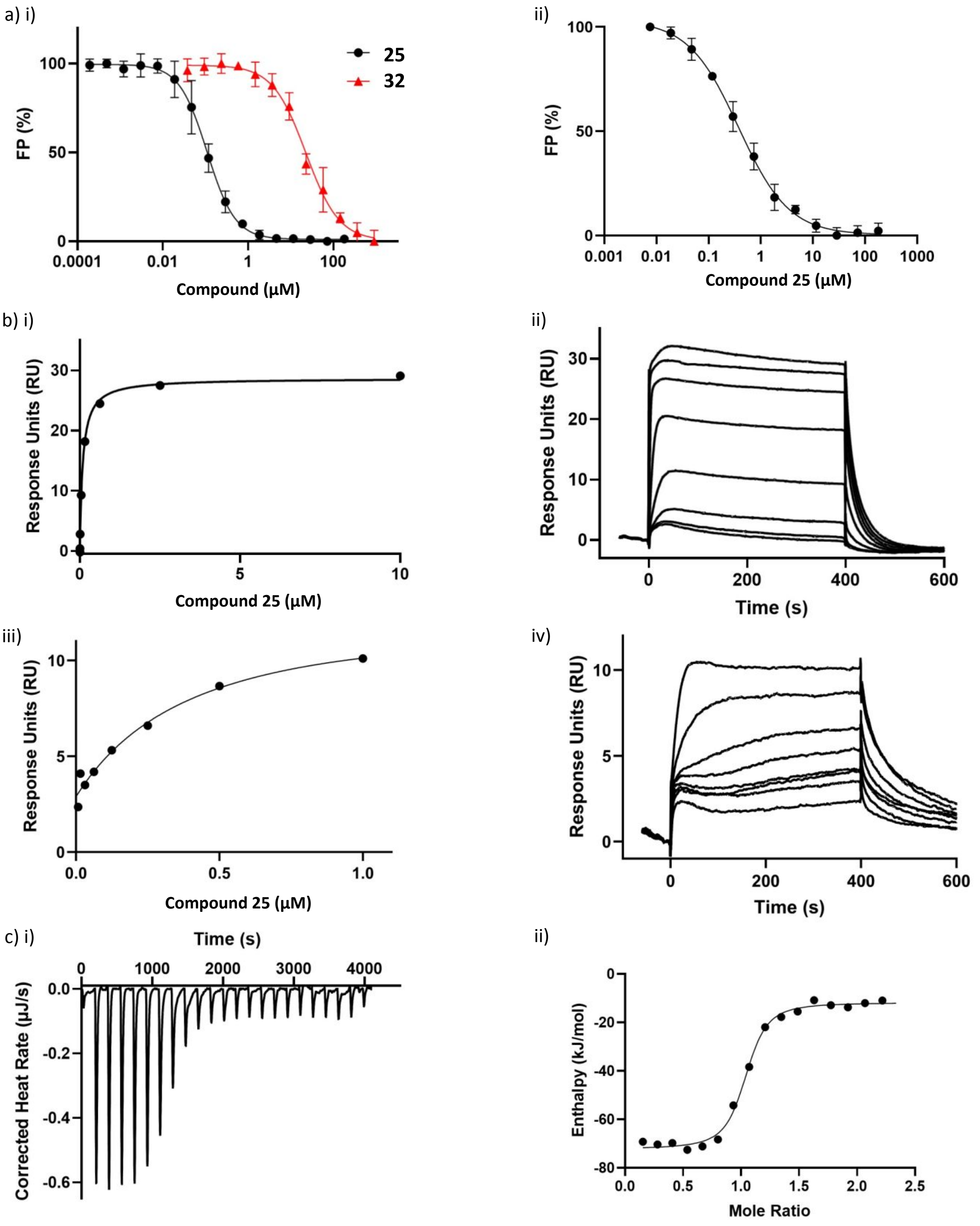

Figure S2. Biophysical characterization of **25** and **32**. a)i) Representative FP competition assay using increasing concentrations of **25** or **32**, a FITC-labeled LRLRGG peptide (50 nM) and

HDAC6-UBD. A K_{disp} of 0.095 ± 0.018 μM, and 33 ± 6.4 μM was obtained from the average of three independent measurements for **25** and **32**, respectively.

Figure S2 continued. ii) Representative FP competition assay using increasing concentrations of **25**, a FITC-labeled LRLRGG peptide (50 nM) and full-length HDAC6. A K_{disp} of 0.26 ± 0.15 μM was obtained from the average of three independent measurements. b) Representative SPR binding i) curve and ii) sensorgram for HDAC6-UBD and **25**. A K_D of 0.084 ± 0.020 μM was obtained from the average of 22 independent measurements. Representative SPR binding iii) curve and iv) sensorgram for full-length HDAC6 and **25**. A K_D of 0.44 ± 0.093 μM was obtained from the average of 11 independent measurements. c) Representative ITC titration and fitted data showing the binding of **25** (100 μM) to HDAC6-UBD (10 μM). A K_D of 0.080 ± 0.023 μM was obtained from the average of three independent measurements.

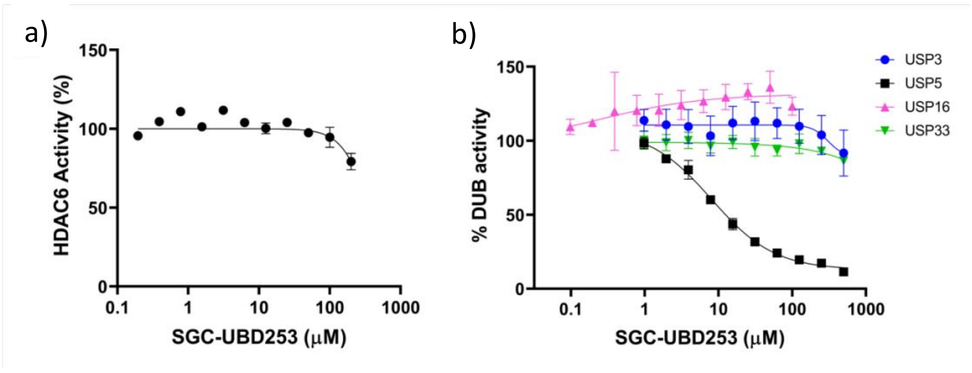

Figure S3. Inhibitory activity of **25**. a) HDAC6 catalytic activity assay shows **25** has no significant effect at concentrations < 100 μM (N=2) b) Ubiquitin rhodamine catalytic activity assay shows **25** does not inhibit USP3, USP16, and USP33 deubiquitinase activity but is inhibitory for USP5 activity (IC_{50}= 8.0 ± 1.1 μM) (N=3).

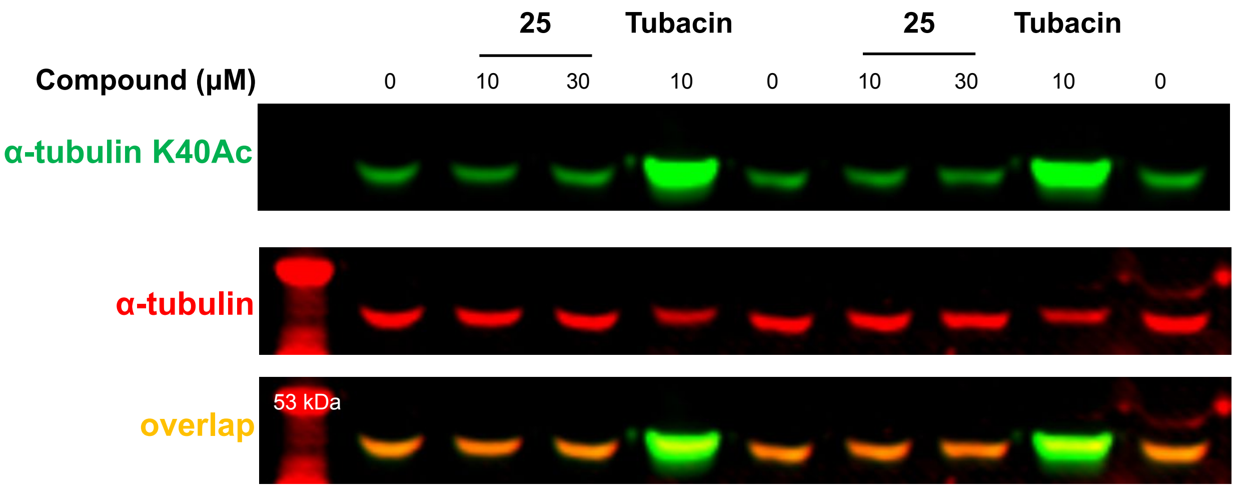

Figure S4. Compound **25** does not inhibit HDAC6 catalytic activity in cells. HEK293T cells were treated with compounds for 24 hours with 10 or 30 μM **25** or 10 μM Tubacin, a catalytic inhibitor of HDAC6.

Synthesis of the biotinylated compound (33)

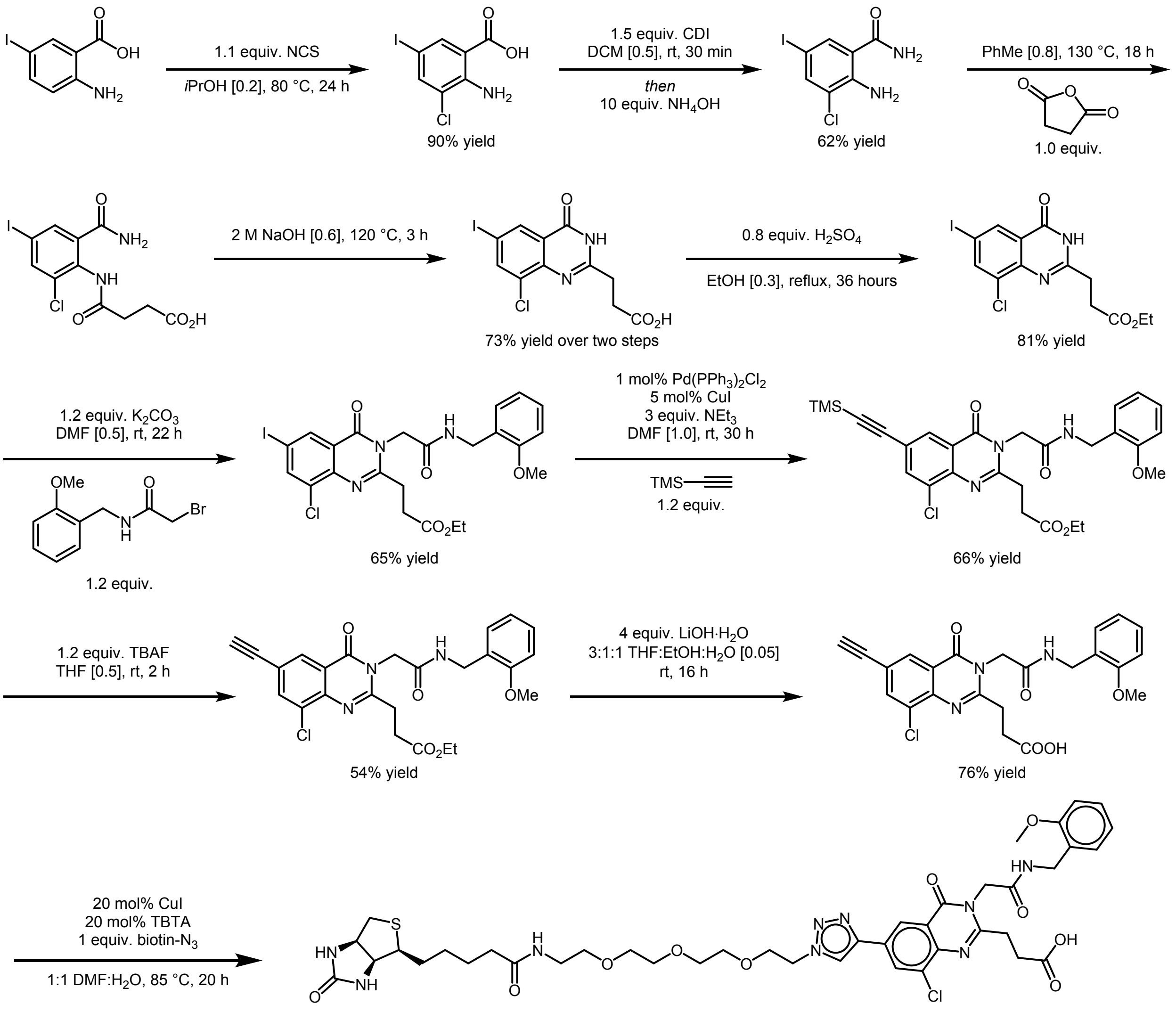

HPLC traces of the compounds 9-32

3-(3-(2-(methylamino)-2-oxoethyl)-4-oxo-3,4-dihydroquinazolin-2-yl)propanoic acid, ***9***

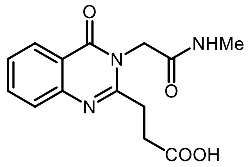

Chemical Formula: $C_{14}H_{15}N_3O_4$
Exact Mass: 289.10626
Molecular Weight: 289.29100

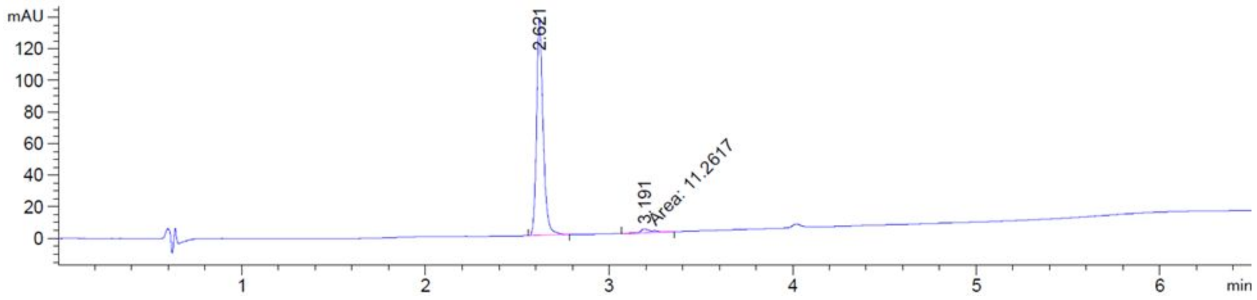

3-(3-(2-(ethylamino)-2-oxoethyl)-4-oxo-3,4-dihydroquinazolin-2-yl)propanoic acid, ***10***

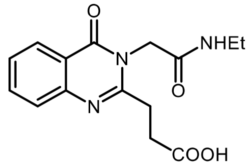

Chemical Formula: $C_{15}H_{17}N_3O_4$
Exact Mass: 303.12191
Molecular Weight: 303.31800

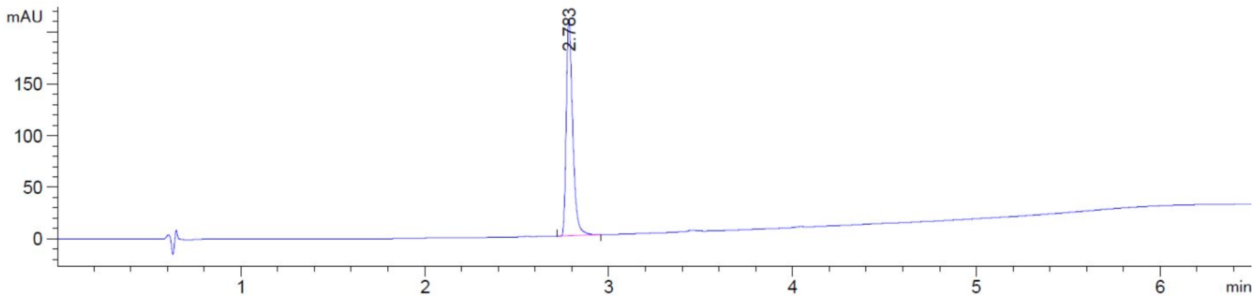

3-(3-(2-(cyclopropylamino)-2-oxoethyl)-4-oxo-3,4-dihydroquinazolin-2-yl)propanoic acid, ***11***

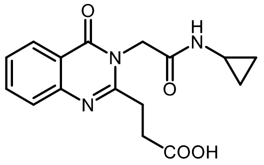

Chemical Formula: $C_{16}H_{17}N_3O_4$
Exact Mass: 315.12191
Molecular Weight: 315.329

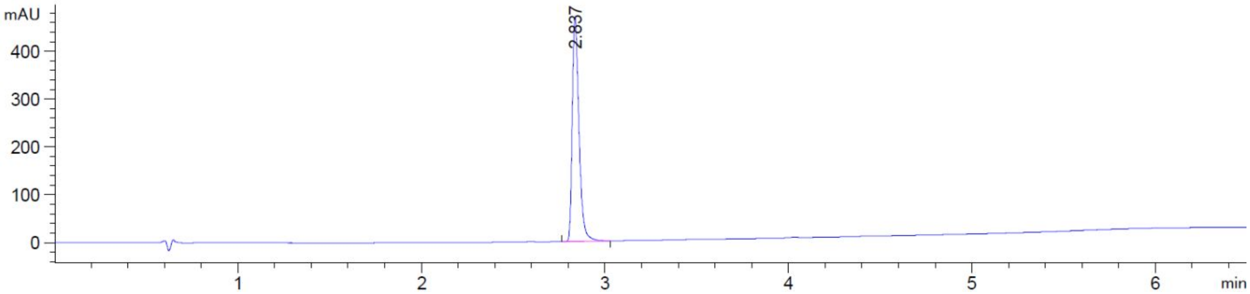

3-(3-(2-(tert-butylamino)-2-oxoethyl)-4-oxo-3,4-dihydroquinazolin-2-yl)propanoic acid, ***12***

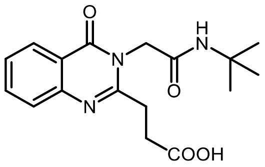

Chemical Formula: $C_{17}H_{21}N_3O_4$
Exact Mass: 331.15321
Molecular Weight: 331.37200

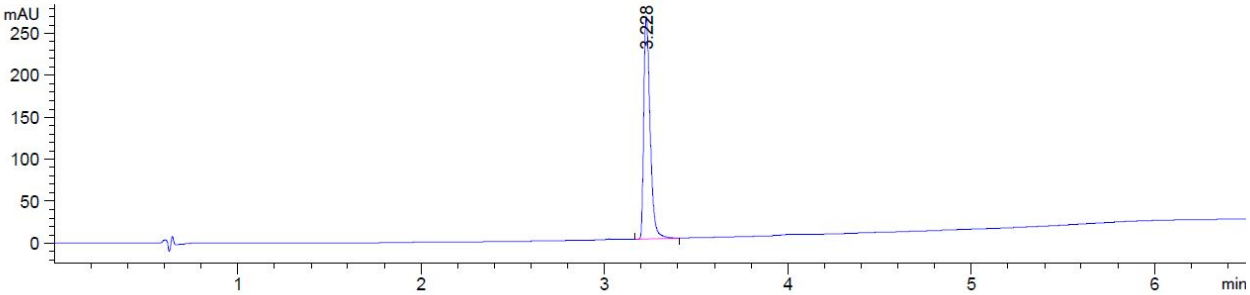

3-(3-(2-(adamantan-1-yl)amino)-2-oxoethyl)-4-oxo-3,4-dihydroquinazolin-2-yl)propanoic acid, ***13***

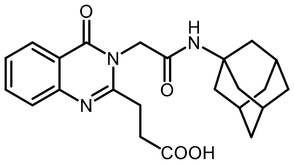

Chemical Formula: $C_{23}H_{27}N_3O_4$
Exact Mass: 409.20016
Molecular Weight: 409.48600

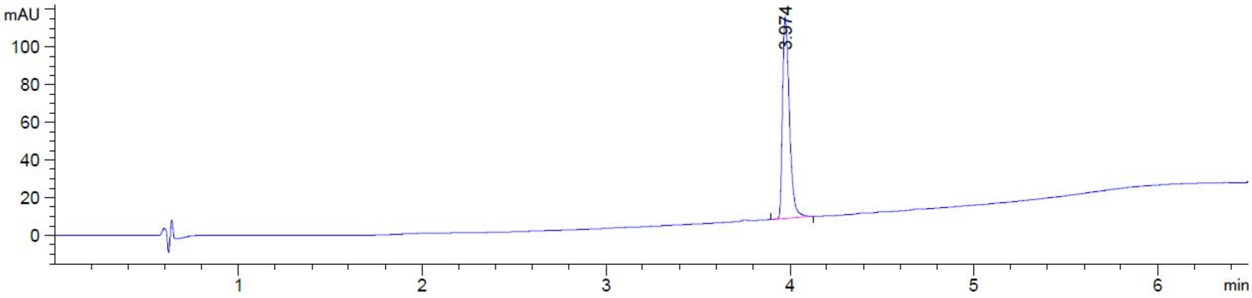

3-(3-(2-((cyclohexylmethyl)amino)-2-oxoethyl)-4-oxo-3,4-dihydroquinazolin-2-yl)propanoic acid, ***14***

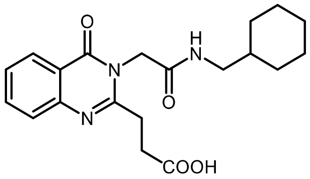

Chemical Formula: $C_{20}H_{25}N_3O_4$
Exact Mass: 371.18451
Molecular Weight: 371.43700

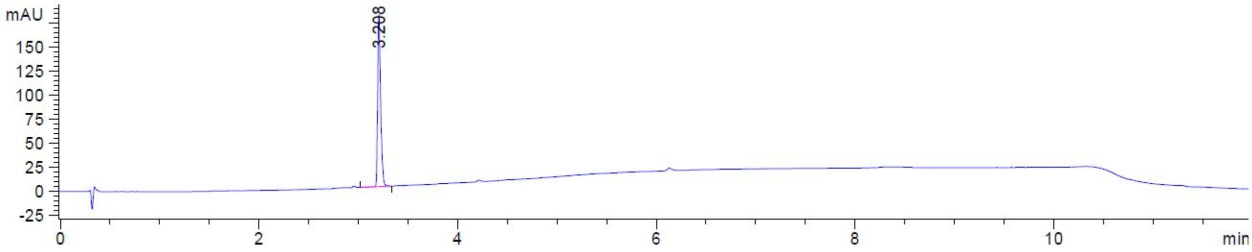

3-(3-(2-(benzylamino)-2-oxoethyl)-4-oxo-3,4-dihydroquinazolin-2-yl)propanoic acid, ***15***

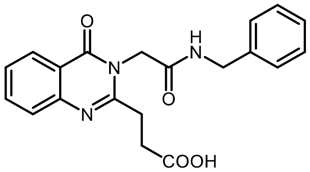

Chemical Formula: $C_{20}H_{19}N_3O_4$
Exact Mass: 365.13756
Molecular Weight: 365.38900

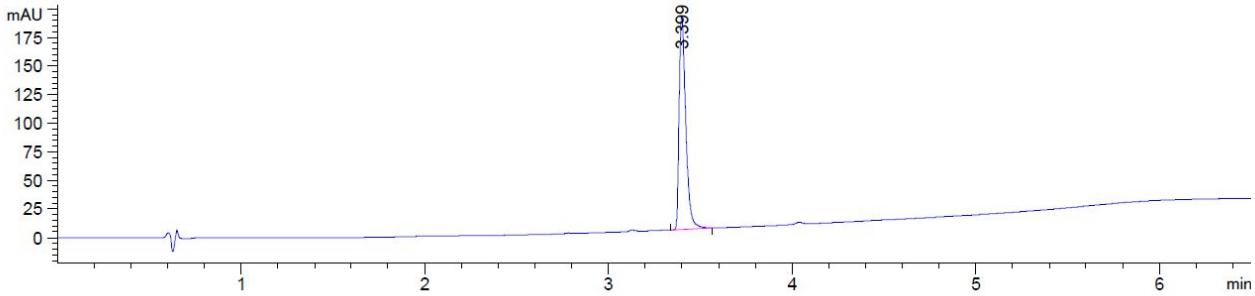

3-(3-(2-((2-methoxybenzyl)amino)-2-oxoethyl)-4-oxo-3,4-dihydroquinazolin-2-yl)propanoic acid, ***16***

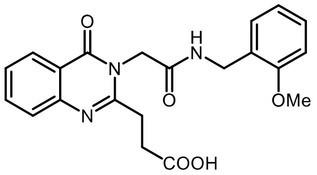

Chemical Formula: $C_{21}H_{21}N_3O_5$
Exact Mass: 395.14812
Molecular Weight: 395.41500

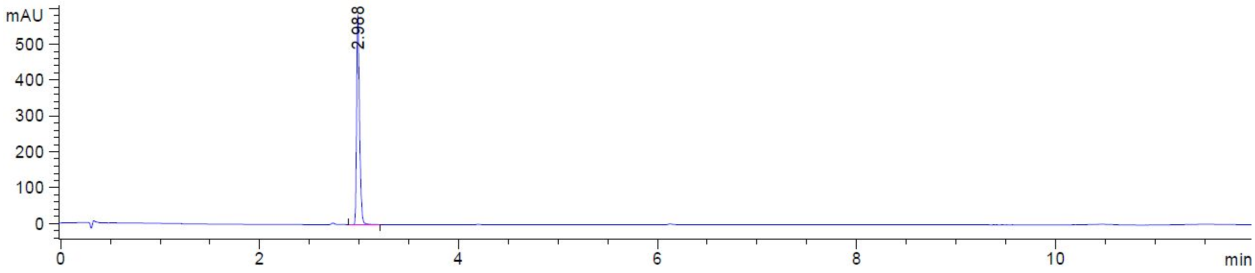

3-(3-(2-((2-chlorobenzyl)amino)-2-oxoethyl)-4-oxo-3,4-dihydroquinazolin-2-yl)propanoic acid, ***17***

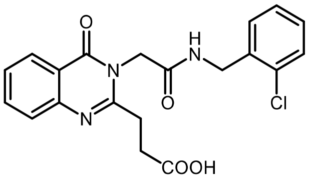

Chemical Formula: $C_{20}H_{18}ClN_3O_4$
Exact Mass: 399.09858
Molecular Weight: 399.83100

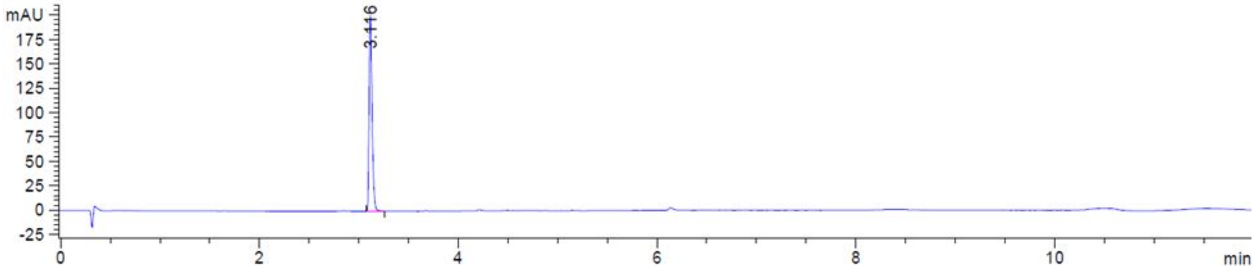

3-(4-oxo-3-(2-oxo-2-((4-(trifluoromethyl)benzyl)amino)ethyl)-3,4-dihydroquinazolin-2-yl)propanoic acid, ***18***

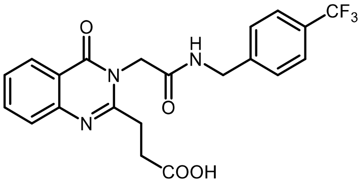

Chemical Formula: $C_{21}H_{18}F_3N_3O_4$
Exact Mass: 433.12494
Molecular Weight: 433.38721

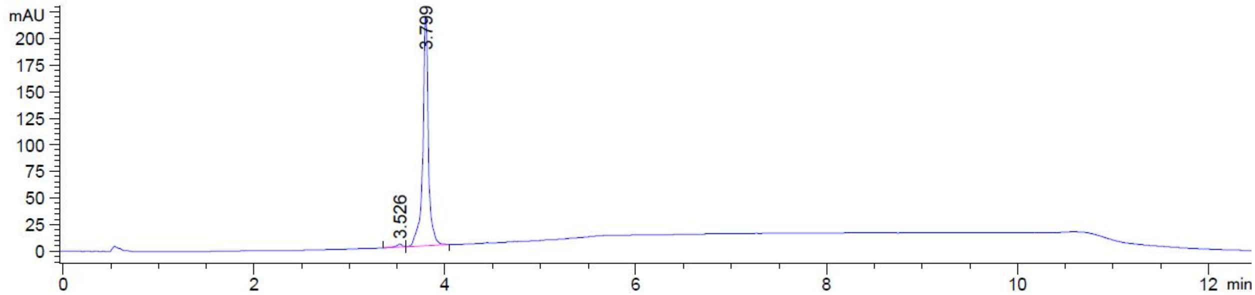

3-(4-oxo-3-(2-oxo-2-((pyridin-3-ylmethyl)amino)ethyl)-3,4-dihydroquinazolin-2-yl)propanoic acid, ***19***

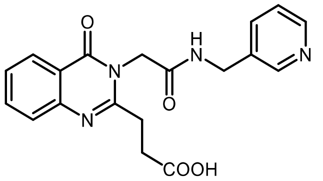

Chemical Formula: $C_{19}H_{18}N_4O_4$
Exact Mass: 366.13281
Molecular Weight: 366.37700

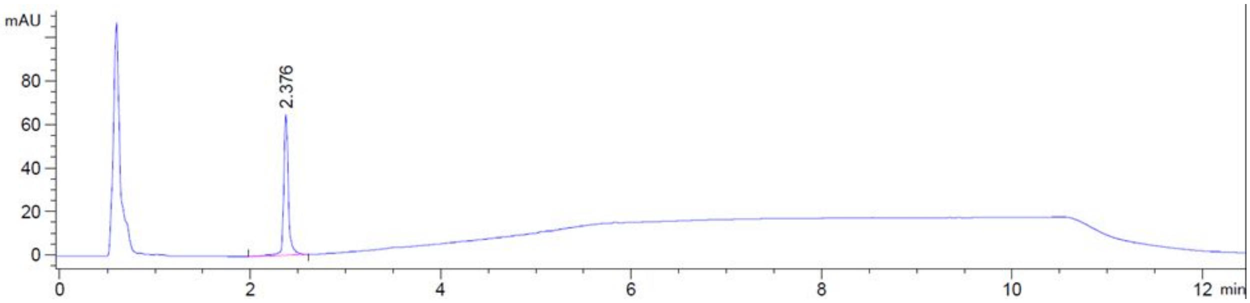

3-(3-(2-((4-methoxybenzyl)amino)-2-oxoethyl)-4-oxo-3,4-dihydroquinazolin-2-yl)propanoic acid, ***20***

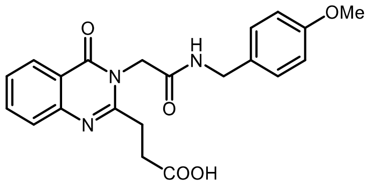

Chemical Formula: $C_{21}H_{21}N_3O_5$
Exact Mass: 395.14812
Molecular Weight: 395.41500

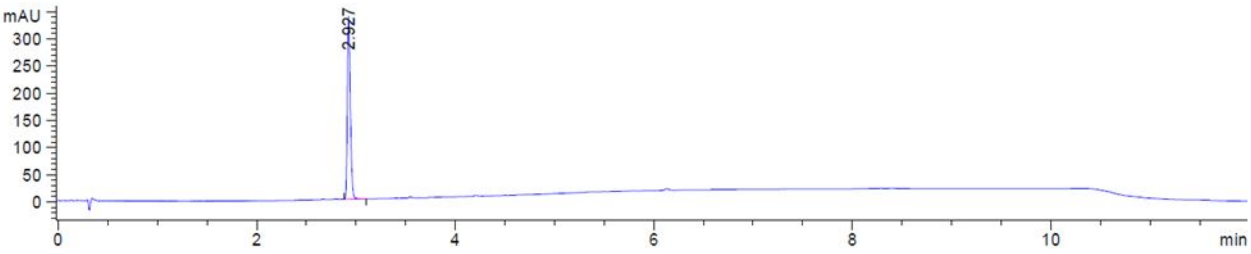

3-(4-oxo-3-(2-oxo-2-(phenethylamino)ethyl)-3,4-dihydroquinazolin-2-yl)propanoic acid, ***21***

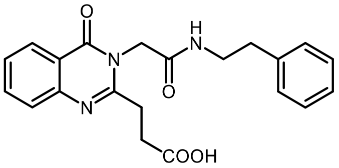

Chemical Formula: $C_{21}H_{21}N_3O_4$
Exact Mass: 379.15321
Molecular Weight: 379.41600

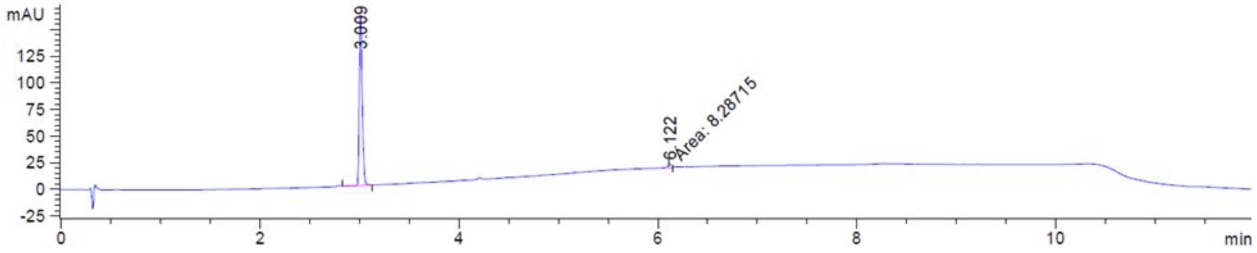

3-(3-(2-((2-methoxyphenethyl)amino)-2-oxoethyl)-4-oxo-3,4-dihydroquinazolin-2-yl)propanoic acid, ***22***

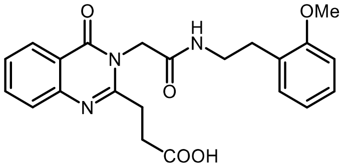

Chemical Formula: $C_{22}H_{23}N_3O_5$
Exact Mass: 409.16377
Molecular Weight: 409.44200

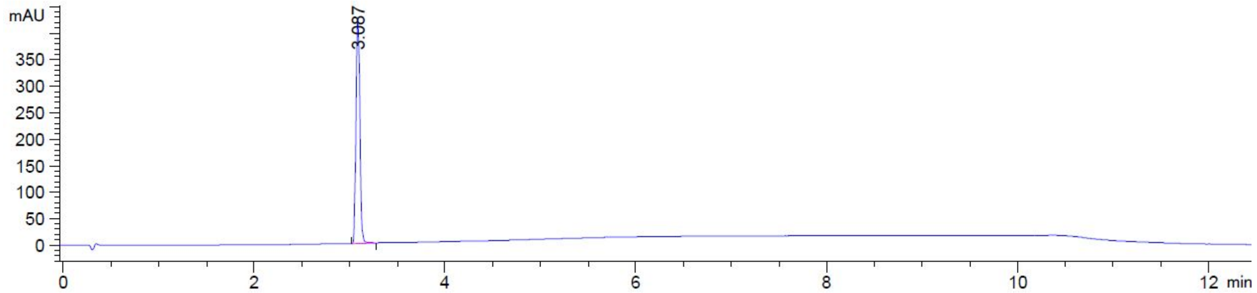

*3-(8-fluoro-3-(2-((2-methoxybenzyl)amino)-2-oxoethyl)-4-oxo-3,4-dihydroquinazolin-2-yl)propanoic acid,**23***

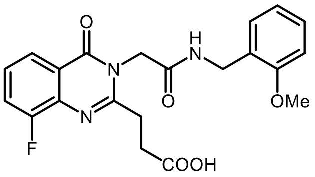

Chemical Formula: $C_{21}H_{20}FN_3O_5$
Exact Mass: 413.13870
Molecular Weight: 413.40540

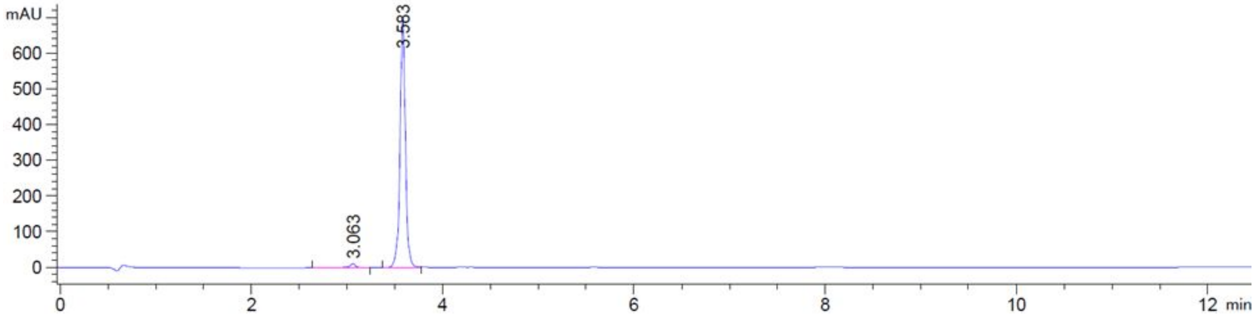

*3-(3-(2-((2-methoxybenzyl)amino)-2-oxoethyl)-8-methyl-4-oxo-3,4-dihydroquinazolin-2-yl)propanoic acid, **24***

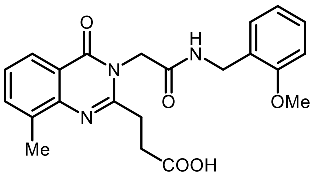

Chemical Formula: $C_{22}H_{23}N_3O_5$
Exact Mass: 409.16377
Molecular Weight: 409.44200

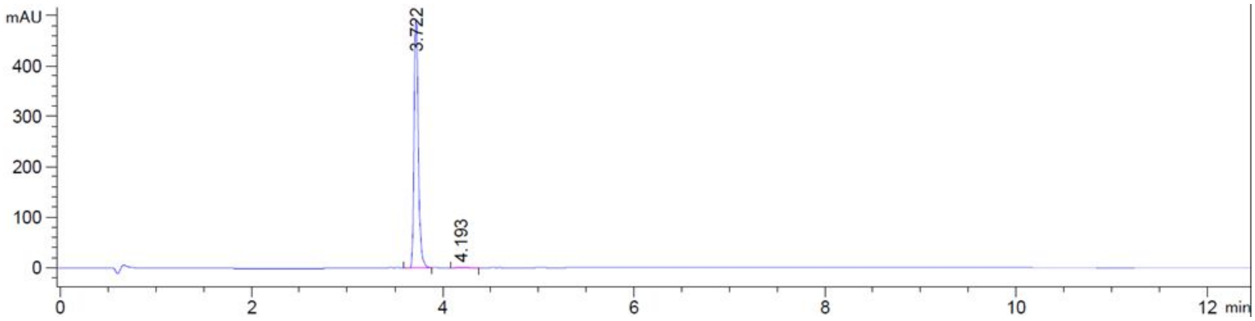

3-(8-chloro-3-(2-((2-methoxybenzyl)amino)-2-oxoethyl)-4-oxo-3,4-dihydroquinazolin-2-yl)propanoic acid, ***25***

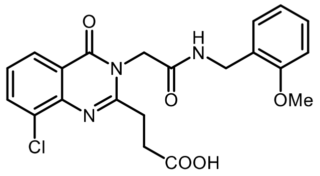

Chemical Formula: $C_{21}H_{20}ClN_3O_5$
Exact Mass: 429.10915
Molecular Weight: 429.85700

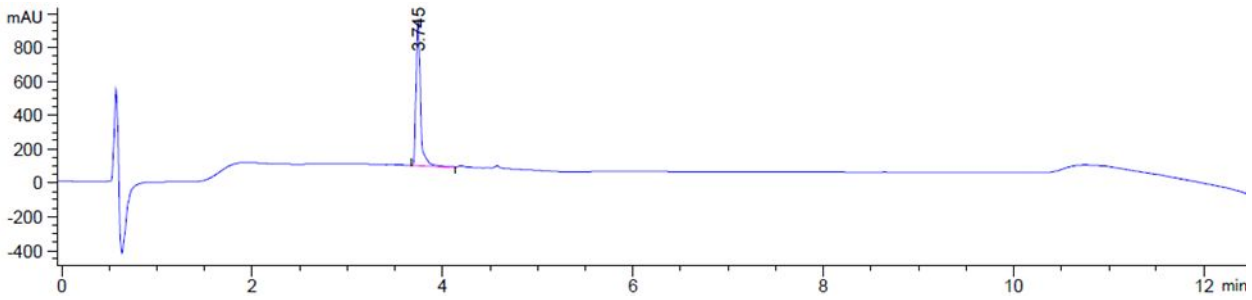

3-(8-chloro-6-iodo-3-(2-((2-methoxybenzyl)amino)-2-oxoethyl)-4-oxo-3,4-dihydroquinazolin-2-yl)propanoic acid, ***26***

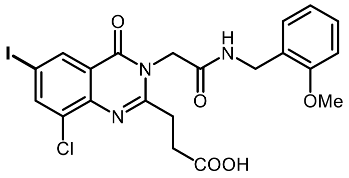

Chemical Formula: $C_{21}H_{19}ClIN_3O_5$
Exact Mass: 555.0
Molecular Weight: 555.8

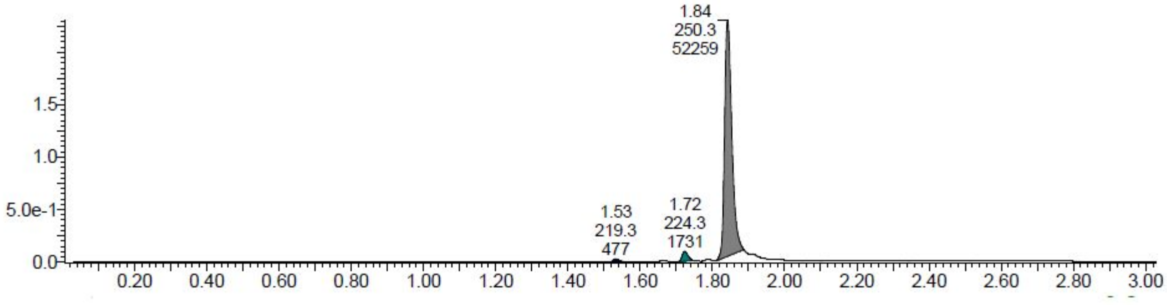

3-(8-chloro-3-(2-((2-methylbenzyl)amino)-2-oxoethyl)-4-oxo-3,4-dihydroquinazolin-2-yl)propanoic acid, ***27***

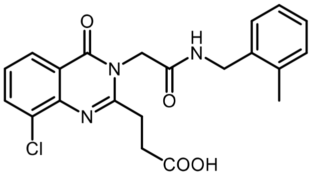

Chemical Formula: $C_{20}H_{17}ClFN_3O_4$
Exact Mass: 417.08916
Molecular Weight: 417.82140

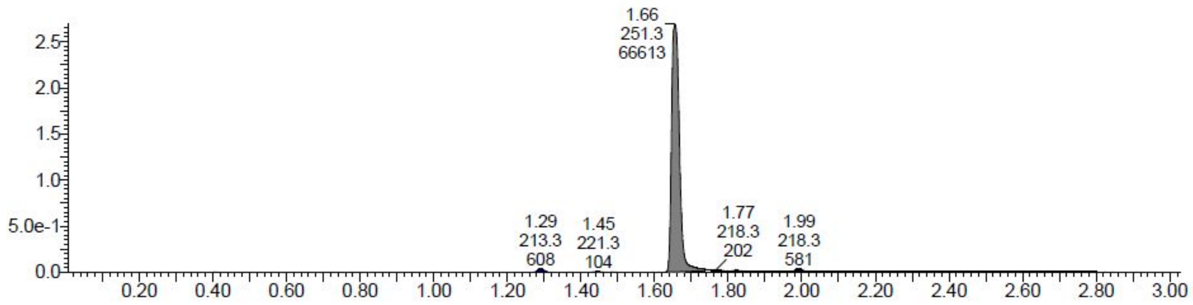

3-(8-chloro-3-(2-((2-fluorobenzyl)amino)-2-oxoethyl)-4-oxo-3,4-dihydroquinazolin-2-yl)propanoic acid, ***28***

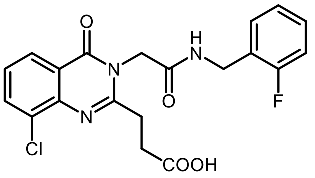

Chemical Formula: $C_{20}H_{17}ClFN_3O_4$
Exact Mass: 417.08916
Molecular Weight: 417.82140

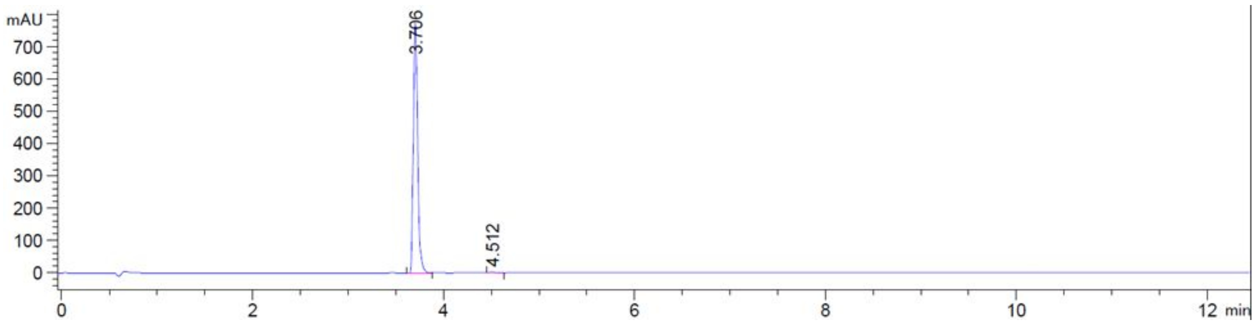

3-(8-chloro-3-(2-((2-chlorobenzyl)amino)-2-oxoethyl)-4-oxo-3,4-dihydroquinazolin-2-yl)propanoic acid, ***29***

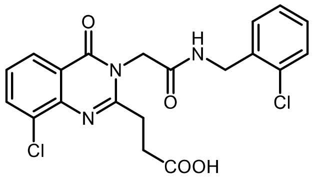

Chemical Formula: $C_{20}H_{17}Cl_2N_3O_4$
Exact Mass: 433.05961
Molecular Weight: 434.27300

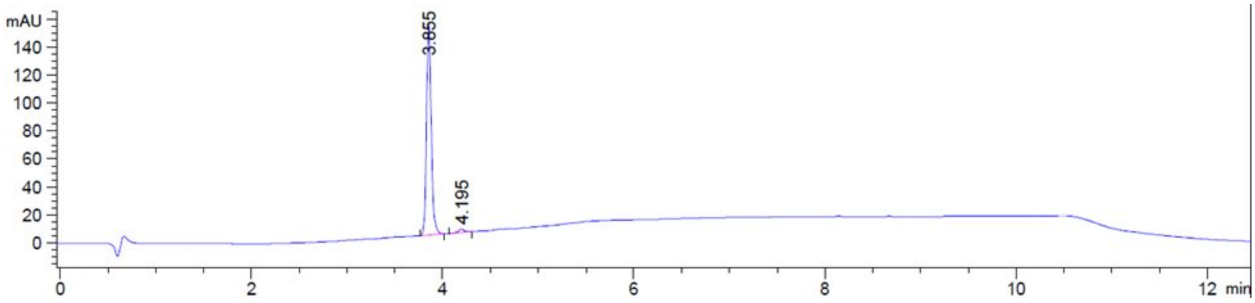

3-(8-chloro-4-oxo-3-(2-oxo-2-(((tetrahydro-2H-pyran-2-yl)methyl)amino)ethyl)-3,4-dihydroquinazolin-2-yl)propanoic acid, ***30***

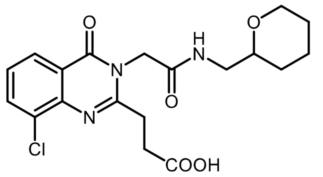

Chemical Formula: $C_{19}H_{22}ClN_3O_5$
Exact Mass: 407.12480
Molecular Weight: 407.85100

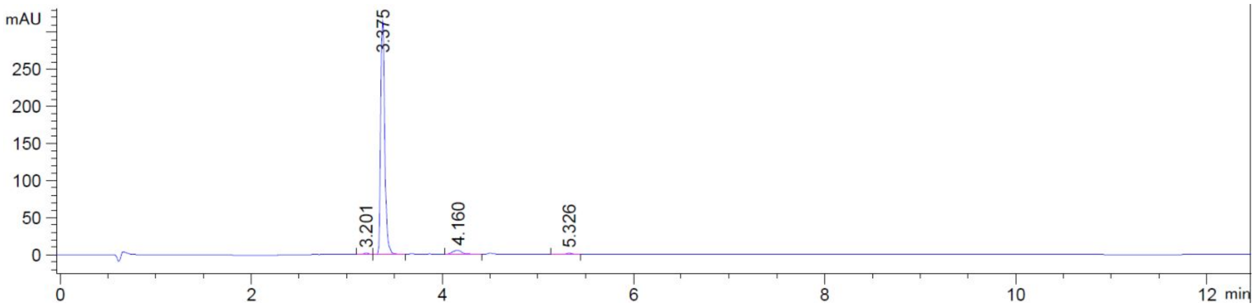

*3-(8-chloro-4-oxo-3-(2-oxo-2-(((tetrahydrofuran-2-yl)methyl)amino)ethyl)-3,4-dihydroquinazolin-2-yl)propanoic acid, **31***

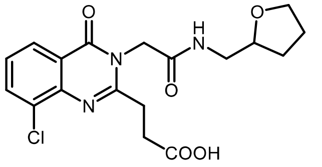

Chemical Formula: $C_{18}H_{20}ClN_3O_5$
Exact Mass: 393.10915
Molecular Weight: 393.82400

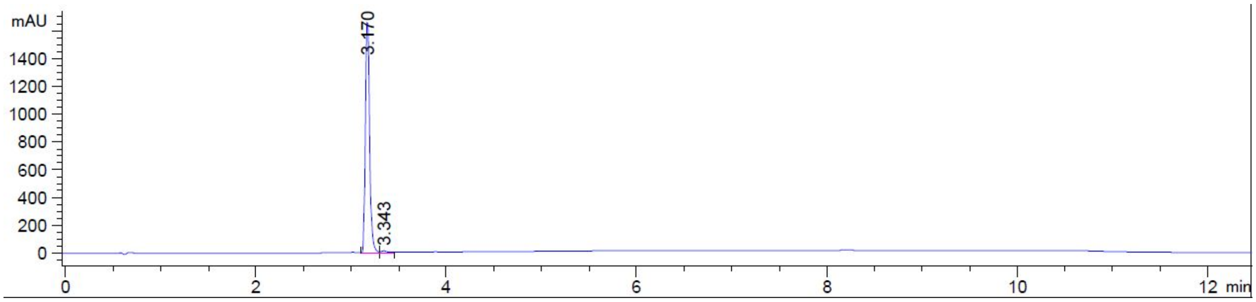

*3-(3-(2-(benzyl(methyl)amino)-2-oxoethyl)-4-oxo-3,4-dihydroquinazolin-2-yl)propanoic acid, **32***

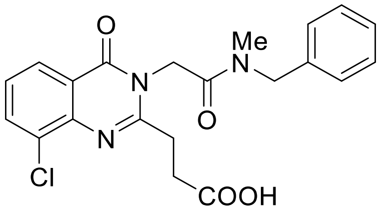

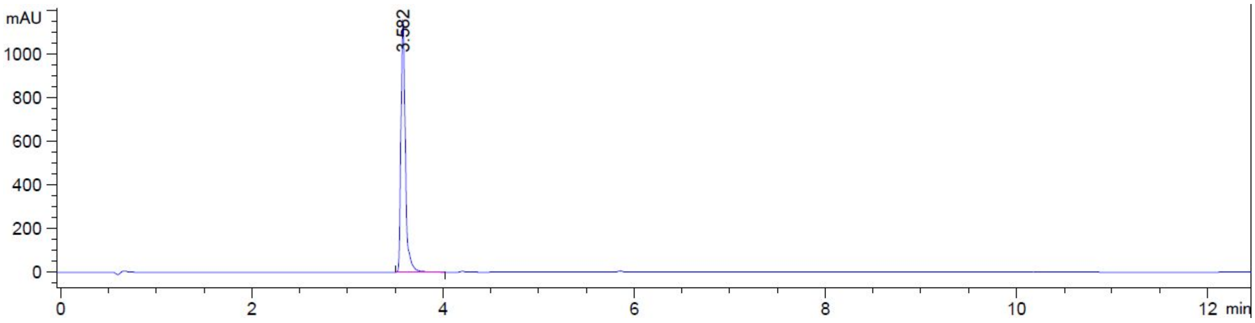

3-(8-chloro-3-(2-((2-methoxybenzyl)amino)-2-oxoethyl)-4-oxo-6-(1-(13-oxo-17-((3aS,4S,6aR)-2-oxohexahydro-1H-thieno[3,4-d]imidazol-4-yl)-3,6,9-trioxa-12-azaheptadecyl)-1H-1,2,3-triazol-4-yl)-3,4-dihydroquinazolin-2-yl)propanoic acid, ***33***

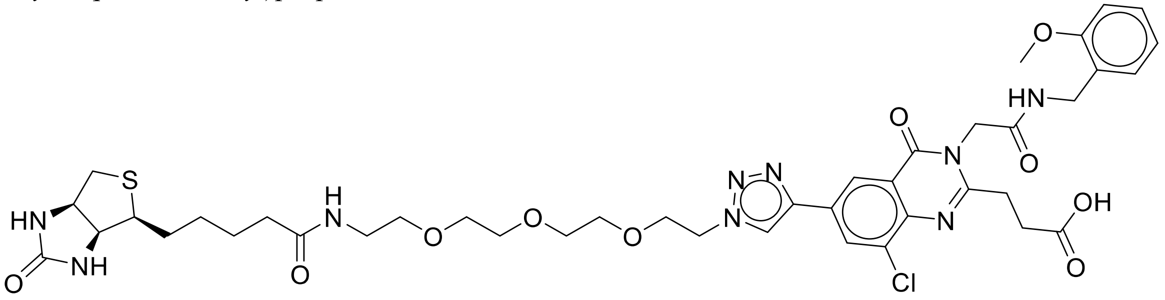

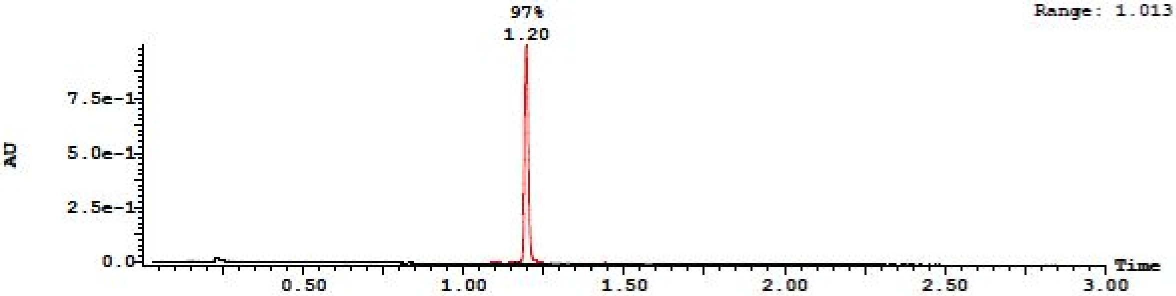

Supplement: Supplementary file 1 — jm3c00314_si_001.pdf [file jm3c00314_si_001.pdf]
